# Twitter-Based Journal Clubs: Additional Facts and Clarifications

**DOI:** 10.2196/jmir.4639

**Published:** 2015-09-16

**Authors:** Joel M Topf, Matthew A Sparks, Francesco Iannuzzella, Edgar Lerma, Thomas Oates, Paul J Phelan, Swapnil Hiremath

**Affiliations:** ^1^ St John Providence Hospital Detroit, MI United States; ^2^ Duke University and Durham VA Medical Centers Durham, NC United States; ^3^ Arcispedale Santa Maria Nuova, IRCCS Reggio Emilia Italy; ^4^ University of Illinois at Chicago/ Advocate Christ Medical Center Oak Lawn, IL United States; ^5^ Imperial College London United Kingdom; ^6^ Royal Infirmary of Edinburgh Edinburgh United Kingdom; ^7^ Ottawa Hospital Research Institute Ottawa, ON Canada

We read the recently published paper on globalization of continuing professional development by Roberts et al with great interest [1]. The authors should be congratulated on their idea as well as their execution of this novel way of evaluating and describing Twitter-based journal clubs. We would like to add to their article by providing some additional advantages and features of a Twitter-based online journal club to provide the reader with a more complete appreciation of their educational potential.

**Figure 1:**
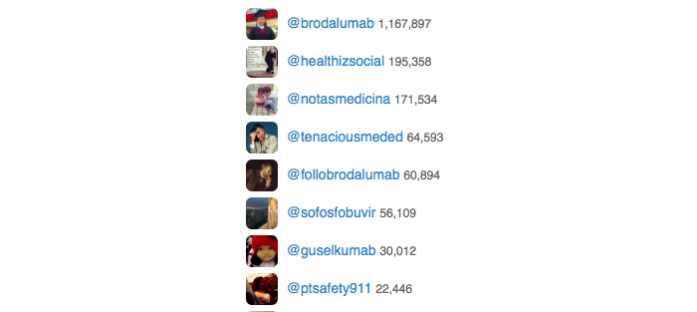
The contribution of 8 "spam" accounts that added 2.4 million impressions to the 15.4 million total, thus falsely inflating the impression count.

First, we would like to caution the authors from relying on *impressions* as tracked by Symplur for two reasons. First, the impression count is the number of tweets multiplied by the number of followers the participant has. This calculation is performed at the time the analytics are generated, not at the time the participant tweeted. So, if participant X has 30 followers and tweets six times this will add 180 impressions to the analytics. If participant X subsequently gains an additional 970 followers, re-running the analytics will now show that participant X was responsible for 6000 impressions. Since users tend to gain more followers over time this makes early journal clubs look more successful than they actually were. Second, a few highly followed accounts can dramatically influence impressions. Today, #NephJC registers 15.4 million impressions, but this includes 2.4 million impressions from 8 spam accounts that tweeted using the #NephJC hashtag to put their message in front of physicians, but did not meaningfully participate in the chat (see Figure 1). Because of these problems, we advise investigators to be cautious when interpreting *impressions* and focus on the other analytics tracked by Symplur. Unfortunately, there is no easy solution to fix this problem. Regular audits of the hashtag could help to identify such accounts. However, this would require one to manually remove promiscuous tweets using the hashtag of interest. 

In the discussion, the authors mention that the "freedom of voluntary participation complicates the establishment of an accurate and efficient record of participation for appropriate ethical acknowledgement for continuing professional development requirements by credentialing authorities." However, registration to a service such as Symplur, allows a Twitter-based journal club to maintain indefinite records of active participation, which we do agree is an essential component of fulfilling continuing professional development requirements by credentialing institutions. 

Twitter-based online journal clubs also provide post-publication peer-review (which in the case of #NephJC is captured in a PubMed Commons comment [2] which links to the pre-chat article summary, a complete transcript and a curated Storify of the chat), which is increasingly recognized as a crucial component of knowledge synthesis and critical review.

We agree that the broad based participation (high number of participants) of the international Urology Twitter Journal Club (#UroJC) is indicative of a successful endeavor. We believe that one of the major reasons driving the high number of participants, apart from its longevity (#UroJC started in October 2012), is that the journal club is not conducted as a focused chat but rather is an open period of discussion stretching over a few days. This allows individuals from many different time zones to contribute at a locally convenient time. In contrast, the live nature of other chats (such as #NephJC) provides a vibrant conversational tone, which likely drives a greater number of tweets, but with the trade-off of limiting participation due to inconvenient timing for various time zones. 

Lastly, regular updates of a systematic review should be performed when new evidence (usually in the form of new studies or trials) becomes available. Referred to as a "living" systematic review, this concept has been encouraged to keep the literature relevant and to narrow the evidence-practice gap [3]. This aspect is perhaps even more relevant to the present review. Between the period that the present authors conducted their search, and the actual publication, another journal club has come into existence (#RheumJC), and the follower count has changed dramatically in some cases (eg #NephJC from 584 to 1360). Interestingly, the National Library of Medicine is encouraging the archiving of online discussions in medicine, such as these journal clubs, and can serve as a useful resource for researchers in this area [4].

Organizing and producing a Twitter-based medical journal club takes a fair amount of time and effort, especially if one takes into account the background work and post-chat summation, active solicitation of participants, and coordination with authors. These efforts are, currently, not tracked or acknowledged by most academic institutions as scholarly activity [5]. Hence, literature, such as this systematic review, are especially welcome since they can help to validate this work as being of scholarly interest.
